# Antifungal Prophylaxis in Immunocompromised Patients

**DOI:** 10.4084/MJHID.2016.040

**Published:** 2016-09-01

**Authors:** Lourdes Vazquez

**Affiliations:** Hospital University of Salamanca (Salamanca, Spain)

## Abstract

Invasive fungal infections (IFIs) represent significant complications in patients with hematological malignancies. Chemoprevention of IFIs may be important in this setting, but most antifungal drugs have demonstrated poor efficacy, particularly in the prevention of invasive aspergillosis.

Antifungal prophylaxis in hematological patients is currently regarded as the gold standard in situations with a high risk of infection, such as acute leukemia, myelodysplastic syndromes, and autologous or allogeneic hematopoietic stem cell transplantation. Over the years, various scientific societies have established a series of recommendations for antifungal prophylaxis based on prospective studies performed with different drugs. However, the prescription of each agent must be personalized, adapting its administration to the characteristics of individual patients and taking into account possible interactions with concomitant medication.

## Introduction

Invasive fungal infections (IFIs) are a leading infectious cause of morbidity and mortality in patients with hematological malignancies,^1^ especially in the contexts of prolonged neutropenia and immunosuppressive treatment. Patients with diseases such as acute leukemia, myelodysplastic syndromes and those undergoing allogeneic hematopoietic stem cell transplant (allo-HSCT) are at major risk of acquiring IFIs.^2^ Their incidence is particularly high in acute myeloid leukemia (AML)^3^,^4^. In some settings, IFIs caused by molds are more frequent than those caused by yeasts, and Aspergillus spp. are the most common pathogens. The risk of invasive aspergillosis (IA) is not constant during all the phases of AML treatment: most AML patients usually experience IA after the first cycle of chemotherapy (first induction), since this is the first time that a colonized patient experiences profound immunosuppression. An IFI during the first induction may dramatically compromise the subsequent therapeutic strategy for AML.^5^,^6^

For this reason, antifungal prophylaxis of IFIs may have a major role in this setting; in the past, chemoprophylaxis with oral polyenes and old triazoles have shown poor efficacy. The availability of new triazoles (e.g., voriconazole, posaconazole), characterized by a wider spectrum, may have modified the role of antifungal prophylaxis in recent times. This review analyzes the efficacy of the various antifungal prophylaxes used over the years.^1^

Scientific societies have established a series of recommendations for antifungal prophylaxis based on prospective studies performed with different drugs.^1^,^7^–^9^ The objective of these recommendations is to create an individualized prescription guideline by each patient’s characteristics.

## Choice of Antifungal Agent for Prophylaxis

Several articles had reviewed the role of the prophylaxis of IFIs in the era before the new antifungals became available.^1^,^5^ Topical therapy with oral polyenes has the potential to prevent candidiasis with less risk of side effects and drug interactions than systemic therapy. It has been found useful for preventing serious Candida infection in high-risk patients. However, this kind of prophylaxis has been disappointing, particularly against Aspergillus.

Some years ago, Uzun and Anaissie^8^ described some criteria to identify the optimal antifungal agent (Table 1): it should be safely administrable over long periods, effective, fungicidal against a broad spectrum of fungal pathogens, inexpensive, available in both oral and intravenous formulations, and associated with a low incidence of resistance. From these criteria, triazoles were identified as a very useful class of oral antifungal drugs, more suitable for chemoprophylaxis of IFIs than AmB and other drugs that are available only in intravenous (iv) formulation.

### Fluconazole

Fluconazole was the first azole systematically used for chemoprophylaxis of IFIs. Due to its high level of systemic activity and low toxicity, fluconazole facilitated an earlier and prophylactic use of systemic antifungals, and it is not contraindicated in patients receiving cyclosporine prophylaxis against graft-versus-host disease (GVHD). However, it is effective only at high doses, under which circumstances it is commonly associated with adverse reactions.^7^–^9^ Fluconazole is active against most Candida strains, although some strains are inherently resistant (e.g., C. krusei and C. glabrata).

### Itraconazole

In contrast to fluconazole, itraconazole is active against Aspergillus spp.^7^,^9^ Two studies have compared the prophylactic activity of these two drugs in hematological patients undergoing allo-HSCT. In the first, itraconazole was administered as an oral solution, and a significant reduction in IFI incidence with no differences in fungal-free survival was observed.^10^ In the second study,^11^ itraconazole was initially administered intravenously and then as an oral solution, resulting in fewer proven IFIs and lower fungal-related mortality, but similar overall mortality, compared with fluconazole after allo-HSCT.^9^ Mild gastrointestinal side effects were observed in the itraconazole arm of both studies.^10^

The study of the GIMEMA-infection group (Gruppo Italiano Malattie Ematologiche dell’Adulto) comparing itraconazole oral solution with placebo found no advantage to itraconazole on the incidence of invasive aspergillosis but did report a significant reduction in candidemia.^11^

The use of itraconazole as prophylaxis is limited by the drug’s poor absorption when given in capsules, and by the gastrointestinal side effects when given as oral suspension.^10^–^11^

## The New Triazoles

### Voriconazole

Voriconazole has been available for clinical use since 2003 and was initially used for the targeted treatment of Aspergillus spp. infections. Some recent clinical trials have tried to demonstrate its additional efficacy in antifungal prophylaxis.^6^,^12^,^13^ In the first study of Vehreschild et al.^6^ a total of 25 AML patients were randomly assigned to receive voriconazole (N=10) or placebo (N=15). The incidence of lung infiltrates until day 21 was 0 (0%) in the voriconazole and 5 (33%) in the placebo group (P=0.06). The average length of stay in hospital was shorter in the voriconazole group (mean 31.9 days) than in the placebo group (mean 37.3 days, P=0.09) ML patients undergoing induction chemotherapy, prophylactic oral voriconazole 200 mg twice daily resulted in trends towards reduced incidences of lung infiltrates and hepatosplenic candidiasis. Voriconazole was safe and well tolerated. Afterward, a multicenter, randomized, double-blind trial compared the ability of fluconazole (n=295) with voriconazole (n=305) for 100 days (180 days in higher-risk cases) to prevent IFIs in patients undergoing myeloablative allo-HSCT.^13^ The authors reported no significant differences in IFI incidence (7.3% vs. 11.2%), and empirical antifungal therapy use (24.1% vs. 30.2%), while fungal-free survival rates (75% vs. 78%) at 180 days and overall survival were similar in fluconazole and voriconazole; however, there were fewer Aspergillus infections in patients treated with voriconazole (9 vs. 17; p=0.05).^12^ The prospective, randomized, open-label, multicenter study by Marks et al.^14^ compared the efficacy and safety of voriconazole (234 patients) with itraconazole oral solution (255 patients) in allo-HSCT recipients. The efficacy of prophylaxis was significantly higher with voriconazole than itraconazole (48.7% vs. 33.2%; p<0.01); itraconazole patients were more likely to receive other systemic antifungals (41.9% vs. 29.9%; p<0.01) but more patients tolerated voriconazole prophylaxis for 100 days (53.6% vs. 39.0%; p<0.01). However, no differences in the incidence of proven/probable IFIs (1.3% vs. 2.1%) and survival to day 180 (81.9% vs. 80.9%) were observed for voriconazole and itraconazole, respectively.^13^

These studies failed to show any significantly greater benefit from voriconazole than from itraconazole or fluconazole in antifungal prophylaxis.^12^,^13^

### Posaconazole

Posaconazole, which has been available for clinical use since 2007, is a new-generation oral azole with in vitro activity against a broad spectrum of medically important fungi, including Candida spp., Aspergillus spp., Zygomycetes, and Fusarium.^14^,^15^

In vitro susceptibility may vary among Zygomycetes and Fusarium species, and there are no in vivo data concerning the efficacy against these rare fungi.^15^ A randomized, multicenter single-blind study conducted by Cornely et al.^7^ evaluated the efficacy and safety of posaconazole (n=304) compared with fluconazole (n=240) and itraconazole (n=58) as prophylaxis for each cycle of chemotherapy (until recovery from neutropenia and complete remission, or for up to 12 weeks) in patients with AML or myelodysplastic syndrome and prolonged neutropenia.^7^ The primary endpoint was the incidence of proven/probable IFIs during treatment, and the secondary endpoints were death from any cause and time to death. With respect to the primary endpoint, proven/probable IFIs were observed in seven patients (2%) of the posaconazole group and 25 patients (8%) of the pooled standard triazole group (absolute reduction in the posaconazole group, −6%; 95% confidence interval, −9.7 to −2.5%; p < 0.001) during the on-treatment period (from randomization to 7 days after the last dose of the study drug). Significantly fewer patients in the posaconazole group had invasive aspergillosis (2 [1%] vs. 20 [7%]; p<0.001). Posaconazole maintained their superiority over pooled standard triazoles in preventing IFIs during the 100-day period after randomization: 14/304 (5%) vs. 33/298 (11%); p=0.003. Posaconazole was also significantly better than pooled standard triazoles, at preventing IA during the treatment phase (2 [1%] vs. 20 [7%]; p<0.001) and during the 100-day period after randomization or over a fixed time period (4 [1%] vs. 26 [9%]; p<0.001). Survival was significantly longer among recipients of posaconazole than among recipients of fluconazole or itraconazole (p=0.04). Serious adverse events possibly or probably related to treatment Cornely^6^ reported by 19 patients (6%) in the posaconazole group and six patients (2%) in the fluconazole or itraconazole group in you study (p=0.01).^6^,^16^

In another randomized, double-blind trial, Ullmann et al.^16^ compared oral posaconazole with oral fluconazole for prophylaxis against IFIs in 600 allo-HSCT recipients with GVHD treated with immunosuppressive therapy. At the end of the fixed treatment (day 112), the difference in incidence of all proven/probable IFIs between posaconazole and fluconazole arms was not significant (5.3% and 9.0%, respectively; p=0.07), but posaconazole was superior to fluconazole in preventing proven/probable IA (2.3% vs. 7.0%; p=0.006). During the exposure period (time from first dose to 7 days after the last dose), posaconazole significantly reduced the incidence of breakthrough proven/probable IFIs (2.4% vs. 7.6%; p=0.004) and IA (1.0% vs. 5.9%; p=0.001) vs. fluconazole. Overall mortality was similar in the two groups, but the number of deaths from invasive fungal infections was lower in the posaconazole group (1%, vs. 4% in the fluconazole group; p=0.046). The incidence of treatment-related adverse events was similar in the two groups, such as the rates of treatment-related serious adverse events (13% and 10%, respectively). Posaconazole proved to be clinically superior to other triazoles in preventing IFIs, especially aspergillosis (table 1). The antifungal agent of choice for the prophylaxis of invasive fungal infection is a triazole (voriconazole or posaconazole). 4,13–17 Itraconazole in oral solution is not considered suitable due its poor digestive tolerance.^10^ However, there is a series of possible metabolic interferences with other drugs that render the use of triazoles unadvisable if there is concomitant treatment with chemotherapy drugs such as vincristine,^18^ immunosuppression with agents such as sirolimus or cyclosporine, QT-prolonging drugs (Table 2), and CYP3A4 activity-inducing drugs (Table 2).^19^,^20^

Another situation in which a triazole may not be the best alternative is the existence of liver function alterations defined by transaminase levels five times the normal value.^17^,^19^,^20^ A triazole is the first prophylactic alternative in the absence of any of these circumstances. Posaconazole has low bioavailability and high interindividual variability. In clinical practice, with a dose of 300 mg/8 h, more than half of the patients do not reach the serum concentration of 700 ng/ml that is considered to be prophylactic.^17^ Therefore, it is convenient to make sure that there are no additional complications that could worsen absorption, such as mucositis, diarrhea, or treatment with antacids or proton pump inhibitors. Moreover, the drug should be given with food, preferably with a high fat content, and carbonated drinks should be avoided.^20^ If these requirements are not met, voriconazole should have priority. If there is any doubt regarding the absorption of posaconazole and its use is considered necessary, the serum concentration should be measured on the third day. A value of >350 ng/ml predicts a serum concentration of >700 ng/ml on the 7th–10th day. If the concentration is <350 ng/ml, it is important to emphasize that the patient should eat fat-rich food and increase the dose to 200 mg/6 h or 400 mg/12 h.^18^,^19^

If, for any of the above reasons (impaired liver function or metabolic interference with other drugs), micafungin or liposomal amphotericin B are alternatives.^18^

### Micafungin

Micafungin is currently the only echinocandin indicated for the prophylaxis of hematological patients.^21^,^22^ In two prospective, randomized, double-blind comparative studies with fluconazole and itraconazole, micafungin at a dose of 50 mg/day was significantly more efficacious than fluconazole (p=0.03) and better tolerated than itraconazole in the prevention of infection by Candida spp. and Aspergillus spp.^23^,^24^ Some authors have used higher doses and intermittently.^24^,^25^ Nevertheless, they do not report differences in dose-related efficacy.^25^,^26^ Micafungin has a high concentration in the alveolar macrophage, which might explain the efficacy of the dose of 50 mg/day.^27^ From the pharmacokinetic, experimental and clinical standpoints, data indicate the possibility of giving doses of 150 mg on alternating days, 200 mg twice a day.

The echinocandins are very active drugs in vitro against Candida and Aspergillus spp. and have demonstrated their efficacy in the prophylaxis and treatment of febrile neutropenia^21^

Micafungin is the most recent echinocandin to be marketed in Spain. It provides better activity against some Candida spp. than other echinocandins do against C. glabrata^21^ and also Aspergillus spp.^21^ It has a low drug interaction potential,^21^,^25^,^26^ which should be relevant in patients requiring concomitant medication, and can be given to those with moderate liver failure when there is any doubt about the use of caspofungin.^21^ Therefore, certain patients could benefit from other therapeutic alternatives. The experience of using micafungin in the treatment of hematological patients has been widely reported.^21^–^27^ Its use has been assessed following the establishment of international guidelines that recommend micafungin in the prophylaxis and empirical treatment of febrile neutropenia.^21^,^27^ In Spain, some centers have accrued experience in the treatment of these patients with micafungin, and we think that it is an appropriate time to describe this experience and evaluate contributions to our specific circumstances.

## Basic Issues

Four basic questions we must plan before starting antifungal prophylaxis, and that we do? That we want to treat fungus? How long do we keep?

First, and perhaps most importantly, it is to select the patient population in which we manage antifungal prophylaxis. In principle, only patients at high or moderate risk of IFI should receive prophylaxis; those at low risk should not (Table 3 ).

However, the German guidelines recommend antifungal prophylaxis in patients at low risk.^1^

Although there have been proposals and validated studies about the definition of IFI risk groups, there is no unanimous agreement. The assignment of the patient to one or other group requires a number of factors to be evaluated a priori for example, the assumption of a certain duration of neutropenia and severity of the mucositis arising from a particular treatment. However, this risk prediction can sometimes be simplistic, as in the case of recipients of allogeneic HSCT.^28^,^29^ This is a group of patients considered to be at high risk, which, in practice, is made up of low-, intermediate- and high-risk subgroups of IFI. In fact, the situation is even more complicated, since a recipient of allogeneic TPH classified pretransplantation as a low-risk young patient, sibling donor with identical HLA and peripheral blood can become, during the transplantation, a patient at high risk of developing GVHD, which requires intense immunosuppression. This example shows that manifests the dynamism of the risk factors for IFI, and may change in the same patient over time.^28^ There have been two large studies of posaconazole prophylaxis. One was carried out in patients with allogeneic HSCT and GVHD,^16^ and the other in 78 patients with neutropenia^7^. It should be noted that in the study of allogeneic HSCT,^8^ posaconazole was not administered during the period of neutropenia after transplantation, but only if the patient had GVHD and needed immunosuppressive treatment, which usually occurs outside the phase of neutropenia. In both studies, posaconazole more efficiently prevented aspergillosis than the comparator (fluconazole or itraconazole). In addition, posaconazole was associated with increased survival in the study of neutropenic patients.^7^ Tolerability of posaconazole was good, being comparable to that of fluconazole. Based on these two studies, posaconazole has become established as the prophylaxis of choice in neutropenic patients and allogeneic HSCT patients suffering from GVHD. Since posaconazole is available in tablet form it can be used in prophylaxis only in those patients who properly tolerate it orally; otherwise, an alternative prophylaxis should be administered.^30^

Experience with micafungin and caspofungin has been reported with respect to candins in prophylaxis. Both require the i.v. administration which limits their use in practice outpatients. Only micafungin has antifungal prophylaxis in HSCT among its technical indications. This indication was based on two large randomized study micafungin versus fluconazole,^23^,^27^ both showing an efficacy equivalent to that of fluconazole and micafungin.

## Amphotericin B Prophylaxis

In general, unless there are contraindications for azoles, the experience with amphotericin i.v. did not support its use prophylactically (Fleming).^4^ However when utilized in the presence of contraindication for triazoles as in patients with acute lymphoid leukemia treated with vincristine, Amphotericin shows its efficacy also in prophylaxis. In allogeneic transplantation, liposomal amphotericin at low dose was well tolerated, but the incidence of invasive fungal infections in patients receiving liposomal amphotericin B was higher than other antifungal agents in the study of Lu Tran,^29^ whereas in the studies of Chaftary and Cordonier.^32^–^33^ High-dose prophylactic liposomal Amphotericin B in HSCT was associated with nephrotoxicity that could be aggravated by the concomitant use of other nephrotoxic agents. On the contrary in patients with acute leukemia in induction, this drug was well tolerated. Better results in patients allotransplanted have been reported by Kargar.^34^ This study rekindled interest in the prophylactic use of liposomal amphotericin and served to increase the level of amphotericin recommendation in the updated ECIL guidelines (Table 3).^35^

## Recommendations

In practice, the selection of these strategies preventing IFI depends on three factors: a) availability of diagnostic techniques necessary for optimal early treatment, such as galactomannan, beta-D-glucan and high-resolution thoracic CT; b) the assessment of the likelihood of IFI for each patient (a partly subjective exercise); and c) the experience of each center (Table 4).^36^ The epidemiology of IFIs can vary between centers and thereby fluency in the type of prophylaxis or type of empirical treatment used.

We must proceed on the basis that there is no single established way to prevent IFI in onco-hematological patients and recipients of TPH. Therefore, the strategies vary among many highly experienced centers. Some do not employ antifungal prophylaxis for filamentous fungi, and its prevention is based on early diagnosis and early treatment, while others emphasize chemoprophylaxis with antifungal filamentous. Both strategies have their advantages and disadvantages.^36^–^38^

Classification into risk groups is proposed (Table 4),^1^,^36^ on the basis of the incidence of expected IFI, building on the proposal of the NCCN (NCCN Clinical Practices guidelines in oncology)^39^ and other guidelines. The 10% cut-off for IFI incidence, above which antifungal chemoprophylaxis is recommended, coincides with that adopted by the ECIL in its recommendations for prophylaxis.^35^

## Figures and Tables

**Table 1:** Antifungal activity.

Species	Antifungal activity							
	FLU	ITR	VOR	POS	CAS	MICA	ANI	AMB
*Candida* species *(69,70, 75)*								
*C. albicans*	*++*	*+++*	*++++*	*++++*	*+++*	*++++*	*++++*	*+++*
*C. parapsilosis*	*++*	*+++*	*+++*	*+++*	*++*	*+*	*++*	*+++*
*C. tropicalis*	*++*	*+++*	*+++*	*+++*	*+++*	*+++*	*++++*	*+++*
*C. glabrata*	*−−*	*++*	*++*	*++*	*+++*	*+++*	*+++*	*+++*
*C. krusei*	*−−*	*+++*	*+++*	*+++*	*+++*	*+++*	*++++*	*++*
*Cryptococcus neoformans (69–72, 76)*	*+*	*+++*	*+++*	*+++*	*−*	*−*	*−*	*+++*
*Var. neoformans*	*+++* 1	*+++*	*+++*	*+++*	*−−*			*+++*
*Var. gattii*	*+++*	*+++*	*+++*	*+++*	*−−*			*+++*
*Aspergillus species (69, 70)*	*−−* 2							
*A. fumigatus*		*+++*	*+++*	*+++*	*+++*	*++++*	*++++*	*+++*
*A. flavus*		*+++*	*+++*	*+++*	*+++*	*++++*	*++++*	*++*
*A. terreus*		*+++*	*+++*	*+++*	*+++*	*++++*	*++++*	*−−*
*A. niger*		*++*	*++*	*+++*	*+++*	*++++*	*++++*	*+++*
*All Zygomycetes (69, 73, 74)*		*−−*	*−−*	*++*				*++*
*Rhizopus species*	*−−*	*++*	*−*	*+++*	*−−*			*+++*
*Mucor species*	*−−* 1	*−*	*−*	*++*	*−−*			*+++*
*Absidia species*		*+++*	*−−*	*+++*				*+++*

Minimum inhibitory concentrations causing 90% inhibition (MIC_90_):++++ ≤ 0.1 μg/mL; +++ ≤ 1.0 μg/mL; ++ ≤ 4.0 μg/mL; + ≤ 8 μg/mL; −> 8 μg/mL; −− ≥ 16 μg/mL. AMB, amphotericin b; ANI, anidulafungin; CAS, caspofungin; FLU, fluconazole; ITR, itraconazole; MICA, micafungin; POS, posaconazole; VOR, voriconazole.

1 MIC_50_ data.

2 Fluconazole MIC_90_ for all molds=256 μg/mL.

**Table 2:** Drugs that prolong QT or induce CYP3A4 significantly.

Drugs prolonging QT	Drugs significantly inducing CYP3A4

Citalopram	Aprepitant
Diphenhydramine	Bosentan
Escitalopram	Carbamazepine
Fluoxetine	Panobinostat
Foscarnet	Phenytoin
Granisetron	Phenobarbital
Macrolides	Rifabutin
Metronidazol	Rifampicin
Nortriptyline	
Ondansetron	
Pentamidine	
Sunitinib	

**Table 3:** Current antifungal prophylaxis for high-risk patients.

Organization/Reference	Strongest evidence-based recommendations
**ECIL 3 (**3**)** **ECIL 4 (**33**)** **European Conference on Infections in Leukemia**	* Allogeneic HSCT, neutropenic phase: fluconazole (400 mg PO/IV QD)/ voriconazole (provisional, 200 mg PO BID); fluconazole (A-I), itraconazole or voriconazole (B-I; therapeutic drug monitoring [TDM] recommended), micafungin (C-I), and liposomal amphotericin B (C-III). Patients aged 13 years or older aerosolized liposomal amphotericin B and posaconazole plus TDM * Allogeneic HSCT, GVHD phase: posaconazole (200 mg PO TID)/ voriconazole (provisional, 200 mg PO BID); posaconazole plus TDM for patients aged 13 years or older (B-I), voriconazole plus TDM for patients aged 2 years or older (B-I), and itraconazole plus TDM (C-II). Other options might include intravenous liposomal amphotericin B and micafungin * Induction chemotherapy of acute leukemia: posaconazole (200 mg PO TID); itraconazole plus TDM (B-I), posaconazole plus TDM in patients aged 13 years or older (B-I), intravenous liposomal amphotericin B (B-II), and fluconazole (C-I; active only against yeasts). Other possible options include aerosolized liposomal amphotericin B, micafungin, and voriconazole plus TDM (no grading)
**NCCN-2012(**39**)** **NCCN-2016 (**40**)** **National Comprhensive Cancer Network**	*ALL Fluconazole/Amphoptericin; Fluconazole or Micafungin Amphotericin B * AML/ MDS with neutropenia: posaconazole; Posaconazolem (category 1) Voriconazolem, Fluconazolem, Micafungin, or Amphotericin B productsn (all category 2B) *Auto HSCT with neutropenia, with mucositis: fluconazole/ micafungin; Fluconazole or Micafungin *Allo HSCT Fluconazole or Micafungin Voriconazole, Posaconazole, Amphotericin B * Significant GVHD: posaconazole; posaconazole; Voriconazole, Echinocandin, Amphotericin B
**IDSA (**29**) Infectious Diseases Society of America**	*Allogeneic HSCT, AML intensive remission-induction or salvage-induction chemotherapy, Candida Prophylaxis with Fluconazole/ itraconazole/ voriconazole/ posaconazole/ micafungin/ caspofungin *AML, MDS intensive therapy, Aspergillus prophylaxis with posaconazole *Allogeneic HSCT, Aspergillus prophylaxis with mold-active drugs in patients with prior invasive aspergillosis
**ASBMT (**28**)** **Am. S. Bone Mar. Tran.**	*Prolonged neutropenia: micafungin (50 mg IV QD) * GVHD: posaconazole (200 mg PO TID)

**Table 4:** Overall risk factors for developing an invasive fungal infection, invasive candidiasis or invasive aspergillosis.

*Risk factor*	*Infection with yeasts*	*Infection with molds*
Underlying disease or condition/HSCT	* AML * ALL * Allogeneic recipients * Mismatched donor increased risk of death from *Candida* infection in ALL	* AML * ALL * Increased risk of IA if hematological malignancy other than in first remission and HSCT recipients * AML patients with previous fungal infection * Allogeneic transplant recipients especially mismatched donors * High risk of IA for the first month (pre-engraftment) after transplant for autologous HSCT recipients * Iron overload * Certain genetic polymorphisms
Neutropenia	* Delayed engraftment * Neutrophils < 0.1×10^9^/ L >3 wk or neutropenia <0.5 × 10^9^ /L > 5 wk	* Increased risk of IA
GVHD	* Acute GVHD	* Moderate-to-severe GVHD grades 2–4 or by chronic GVHD
Steroid use (to treat GVHD)	* Steroid use > 2 mg/kg >2 wk or > 1 mg/kg > 1 wk if ANC < 1 × 10^9^ /L > 1 wk * ALL	* Risk of IA increased 2.1-fold * Steroid use plus development of moderate-to-severe GVHD 33% probability of IA * ALL
Age	* Extremes of age (< 1 and > 70 years) * Increasing risk by decade in patients undergoing HSCT	* Age > 40 years increases the risk of IA in patients undergoing HSCT.
Other	* Initial treatment or GVHD with cyclosporine FTBI plus cyclophosphamide (99), broad- spectrum antimicrobials (9) or indwelling catheters * High-dose cytarabine/ etoposide/ daunorubicin	* CMV infection
Non-LAF		* Increased risk of IA

ALL, acute lymphocytic leukemia; AML, acute myeloid leukemia; ANC, absolute neutrophil; CMV, cytomegalovirus; FTBI, fractionated total body irradiation; GVHD, graft-versus-host disease; HSCT, hematopoietic stem cell transplantation; IA, invasive aspergillosis; non-LAF, transplant outside of laminar airflow room. Adapted by Cornely OA et al.^36^
